# Timing of intubation and ICU mortality in COVID-19 patients: a retrospective analysis of 4198 critically ill patients during the first and second waves

**DOI:** 10.1186/s12871-023-02081-5

**Published:** 2023-04-27

**Authors:** Sara Manrique, Laura Claverias, Mónica Magret, Joan Ramón Masclans, María Bodi, Sandra Trefler, Laura Canadell, Emili Díaz, Jordi Sole-Violan, Elena Bisbal-Andrés, Ruth González Natera, Antonio Albaya Moreno, Montserrat Vallverdu, Juan Carlos Ballesteros, Lorenzo Socias, Federico Gordo Vidal, Susana Sancho, Ignacio Martin-Loeches, Alejandro Rodriguez

**Affiliations:** 1 Critical Care Department – Hospital Universitario de Tarragona Joan XXIII, Mallafre Guasch 4, Tarragona, 43005 Spain; 2 Rovira i Virgili University, Tarragona, Spain; 3 URV/IISPV, Tarragona, Spain; 4 Critical Care Department – Hospital del Mar, Barcelona, Spain; 5 Pharmacy Department – Hospital Universitario de Tarragona Joan XXIII, Tarragona, Spain; 6 Critical Care Department – Hospital Parc Tauli, Sabadell, Spain; 7 Critical Care Department – Hospital Dr. Negrin, Las Palmas de Gran Canaria, Spain; 8 Universidad Fernando Pessoa- Canarias, Las Palmas, Spain; 9 Critical Care Department Hospital – Arnau de Vilanova, Valencia, Spain; 10 Critical Care Department – Hospital Militar de Zaragoza, Zaragoza, Spain; 11 Critical Care Department Hospital de Guadalajara, Guadalajara, Spain; 12 Hospital Arnau de Vilanova, Lleida, Spain; 13 Hospital Clinico Salamanca, Salamanca, Spain; 14 Critical Care Department Hospital – Hospital Son Llatzer, Palma de Mallorca, Spain; 15 Critical Care Department Hospital –Hospital de Henares, Coslada, Spain; 16 Critical Care Department Hospital –Hospital Universitari i Politènic la Fe, Valencia, Spain; 17 Department of Intensive Care Medicine, Multidisciplinary Intensive Care Research Organization (MICRO), St. James’s Hospital, Dublin, Ireland

**Keywords:** SARS-COV2, COVID-19 pneumonia, Timing to intubation, Mechanical ventilation

## Abstract

**Background:**

The optimal time to intubate patients with SARS-CoV-2 pneumonia has not been adequately determined. While the use of non-invasive respiratory support before invasive mechanical ventilation might cause patient-self-induced lung injury and worsen the prognosis, non-invasive ventilation (NIV) is frequently used to avoid intubation of patients with acute respiratory failure (ARF). We hypothesized that delayed intubation is associated with a high risk of mortality in COVID-19 patients.

**Methods:**

This is a secondary analysis of prospectively collected data from adult patients with ARF due to COVID-19 admitted to 73 intensive care units (ICUs) between February 2020 and March 2021.

Intubation was classified according to the timing of intubation. To assess the relationship between early versus late intubation and mortality, we excluded patients with ICU length of stay (LOS) < 7 days to avoid the immortal time bias and we did a propensity score and a cox regression analysis.

**Results:**

We included 4,198 patients [median age, 63 (54‒71) years; 71% male; median SOFA (Sequential Organ Failure Assessment) score, 4 (3‒7); median APACHE (Acute Physiology and Chronic Health Evaluation) score, 13 (10‒18)], and median PaO_2_/FiO_2_ (arterial oxygen pressure/ inspired oxygen fraction), 131 (100‒190)]; intubation was considered very early in 2024 (48%) patients, early in 928 (22%), and late in 441 (10%). ICU mortality was 30% and median ICU stay was 14 (7‒28) days. Mortality was higher in the “late group” than in the “early group” (37 vs. 32%, *p* < 0.05). The implementation of an early intubation approach was found to be an independent protective risk factor for mortality (HR 0.6; 95%CI 0.5‒0.7).

**Conclusions:**

Early intubation within the first 24 h of ICU admission in patients with COVID-19 pneumonia was found to be an independent protective risk factor of mortality.

**Trial registration:**

The study was registered at Clinical-Trials.gov (NCT04948242) (01/07/2021).

**Supplementary Information:**

The online version contains supplementary material available at 10.1186/s12871-023-02081-5.

## Background

Patients with coronavirus disease 2019 (COVID-19), caused by the SARS-CoV-2 virus, can develop severe hypoxemia and acute respiratory distress syndrome (ARDS) [1]. Delayed intubation in ARDS patients has been reported to increase mortality, so ARDS guidelines recommend early recognition of acute respiratory failure (ARF) and intubation [2]. At the beginning of the pandemic when ARF was labelled as ARDS, the indication was to immediately intubate [3-7]. Subsequently, it was observed that these patients could tolerate even a severe degree of hypoxemia without or seldom respiratory symptoms. These issues, as well as the lack of ventilators and ICU rooms, resulted in patients being intubated later. So, it was observed that some of these patients never ended up intubated, and potential secondary effects associated with invasive mechanical ventilation (IMV), as ventilator-induced lung injury (VILI) and ventilator-associated pneumonia (VAP) were avoided [8, 9]. Published COVID-19 clinical practice guidelines recommended a trial of non-invasive respiratory support first [10].

Nowadays, whether to intubate early or late remains controversial. Several expert consensus recommend early intubation in patients with a severe presentation of COVID-19 pneumonia because a large proportion of these patients could potentially develop ARF requiring emergency orotracheal intubation, which is associated to a higher risk of viral transmission to healthcare workers [3-6]. Moreover, some documents also recommended early intubation to prevent patient-self-induced lung injury (P-SILI) [11, 12]. On the other hand, there is probably not enough evidence for P-SILI in NIV and obviously there are many uncertainties regarding the right strategy to recommend the use of an invasive or not mechanical ventilation in patients with COVID-19 pneumonia [13].

A recent meta-analysis including more than 8,000 critically ill patients with COVID-19 suggested that the timing of intubation may have no impact on mortality [14]. This study acknowledged the huge heterogeneity of the studies analysed, that reflects different clinical strategies regarding this topic and it would preclude definitive conclusions. We hypothesized that delayed intubation is associated with higher mortality in COVID-19. To test this hypothesis, we aimed to determine the association of intubation timing on ICU mortality in a large population of critically ill COVID-19 patients.

## Methods

### Design

This a secondary analysis of prospectively collected data from adult patient with ARF due to COVID-19 admitted to 73 intensive care units (ICUs) (71 in Spain, 1 in Andorra, and 1 in Ireland) between February 22, 2020 and March 11, 2021. Data were retrieved from the Spanish Society of Intensive Care Medicine and Coronary Units’ (SEMICYUC) registry of COVID-19 patients. The study was registered at Clinical-Trials.gov (NCT04948242) (01/07/2021).

We selected all consecutive patients ≥ 15 years old who met the criteria for COVID-19 pneumonia and ARDS according to the Berlin criteria [15]. We excluded patients with limitations on life support and those with missing data. Patients were followed up to ICU discharge or death.

### Data collection

Patients’ demographic and clinical data were recorded on a case report form, anonymized, and sent to the coordinating centre, where all the information was entered in the COVID-19 registry, as reported elsewhere [16]. Demographic characteristics (age, sex, and body mass index), comorbidities, laboratory tests, microbiologic results, radiological findings, time to intubation, non-invasive respiratory support (oxygen, high-flow nasal cannula [HFNC], non-invasive ventilation [NIV], and IMV) at ICU admission and 24 h after admission, complications, organ-support measures, treatments administered, and outcomes were registered. Disease severity was evaluated 24h after ICU admission using the APACHE II score and SOFA score.

Clinical decisions (e.g., indicating intubation, respiratory support, and treatments) were not protocolized and were left to the discretion of attending physicians. COVID-19 diagnosis was confirmed by reverse transcription-polymerase chain reactions (rt-PCR) for SARS-CoV-2 and COVID-19 pneumonia was diagnosed through clinical signs of pneumonia with acute respiratory failure and lung infiltrates on chest imaging [17].

### Outcome

The primary outcome was all-cause ICU mortality.

Secondary outcomes included hospital mortality, ICU and hospital LOS, and time under IMV.

### Analysis plan

We classified intubation time as 1) very early: before or at ICU admission; 2) early: < 24 h after ICU admission, 3) late: > 24 h after ICU admission and 4) never intubated. We compared early versus late groups. We first displayed the clinical characteristics of the patients using the very early group as a reference group since these group patients showed clear signs of ARF and emergency orotracheal intubation. Therefore, our main analysis will focus on patients who were intubated within the first 24 h of ICU admission compared to those who were intubated after this time period.

### Statistical analysis

No statistical sample size calculation was performed, and sample size was equal to the number of patients admitted to the participating ICUs during the study. Categorical variables were expressed as frequencies and percentages, and continuous variables were expressed as medians and first and third quartiles (Q1-Q3). To compare baseline characteristics between groups, we used chi-square or Fisher’s exact tests for categorical variables and the Mann-Whitney U for continuous variables. All tests were two-tailed, and significance was set at *p* < 0.05.

To investigate possible hospital-level or inter-hospital variation in ICU mortality (as random effects), we used multilevel logistic regression analysis with a conditional random intercept model [16], according to the total number of beds in each hospital (< 200, 200−500, or > 500). Regression coefficients were summarized as the variance with standard deviation and the interclass correlation coefficient (ICC).

All patients were classified as intubated within the first day of ICU stay or thereafter. The first time recorded for the purpose of the study was the day of ICU admission, but all patients were censored within the first 7 days after ICU admission and were discarded to avoid immortal time bias. Propensity score (PS) using the genetic matching algorithm was used to reduce selection bias and balance the covariance matrix of both groups. The match was 1:1 with replacement and ties, and no calibrators. The variables selected for inclusion in the matching model were the variables related to outcome. These variables included demographic characteristics and comorbidities, disease severity and laboratory data.

To investigate the association between baseline (ICU admission) variables and early or late intubation, a multivariate analysis (binary logistic regression) was performed. The multivariate model comprised factors of clinical interest and all significant covariates in the univariate analysis. The results are presented as odds ratios (OR) and 95% confidence intervals (CI).

Finally, to confirm the results, a survival analysis (Cox hazard regression) was performed to investigate whether survival time was related to covariates, and to estimate the effect size of intubation timing and ICU mortality in the PS matched cohort. The results are presented as hazard ratio (HR) and 95% confidence intervals (CI).

SPSS version 24 (IBM Corp. Armonk, NY, USA) and R software (cran.r-project.org) were used for the data analyses.

## Results

### Whole population

**Fig. 1:**
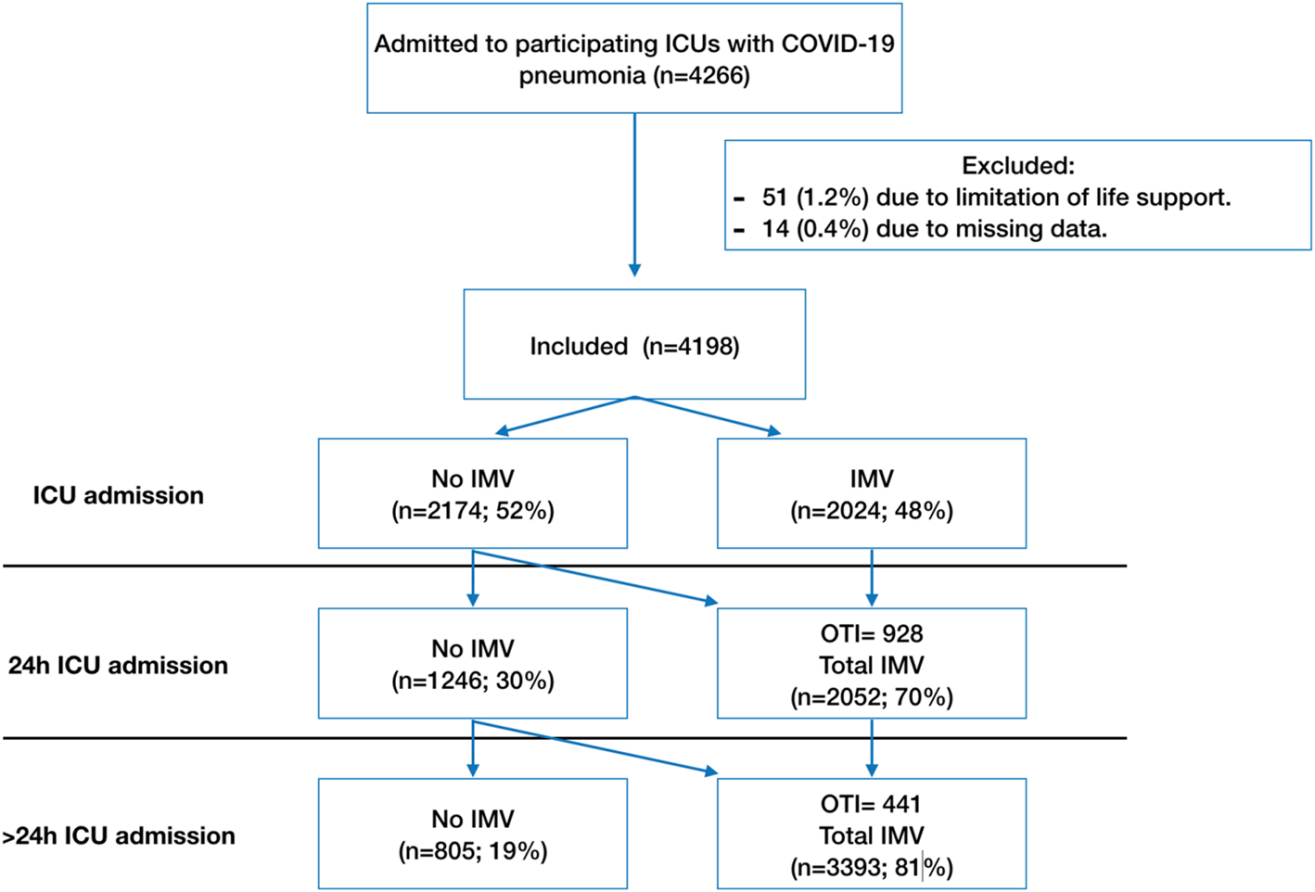
Flowchart of the study. ICU: Intensive Care Unit, IMV= Invasive mechanical ventilation, OTI= Orothraqueal intubation

During the study period, 4,266 patients were admitted to the participating ICUs with COVID-19 pneumonia; 51 (1.2%) were excluded from the analyses due to end-of-life decisions during their ICU stay and 17 (0.4%) were excluded due to missing data (Fig. 1). Thus, we analyzed data from 4,198 patients [median age, 63 (54‒71) years; 71% men; median SOFA score, 4 (3‒7), median APACHE II score, 13 (10‒18); and median PaO2/FiO2 ratio at ICU admission, 131 (90‒190)] (Table 1).

**Table 1:** General characteristics of the population

**Variable**	**Entire population (*N* = 4198)**	**Very early group (*N* = 2024)**	**Early group (*N* = 928)**	**Late group (*N* = 441)**	**Never intubated (*N* = 805)**
***Demographics and severity of illness***					
**Male, n (%)**	2971 (71)	1436 (71)	670 (72)	317 (72)	548 (68)
**Age, in years, median (p25-75)**	63 (54‒71)	65 (57‒72)	64 (56‒71)	62 (50‒70)	57 (48‒66)
**SOFA, median (p25‒75)**	4 (3‒7)	6 (4‒8)	4 (3‒7)	3 (2‒6)	3 (2‒4)
**APACHE II, median (p25‒75)**^**a**^	13 (10‒18)	15 (11‒20)	14 (10‒17)	13 (10‒16)	10 (7‒13)
***Comorbidities***					
**Hypertension, n (%)**	1931 (46)	980 (48)	466 (50)	175 (40)	310 (39)
**Obesity (> 30 kg/m**^**2**^**), n (%)**	1508 (36)	739 (36)	354 (38)	150 (34)	265 (33)
**Diabetes, n (%)**	949 (23)	458 (23)	262 (28)	85 (19)	144 (18)
**Chronic heart failure, n (%)**	139 (3)	68 (3)	32 (3)	18 (4)	21 (3)
**Chronic lung disease, n (%)**	286 (7)	162 (8)	69 (7)	24 (5)	31 (4)
**Asthma, n (%)**	262 (6)	123 (6)	60 (6)	26 (6)	53 (7)
**Ischemic heart disease, n (%)**	256 (6)	128 (6)	64 (7)	18 (4)	37 (5)
**Immunosuppression, n (%)**	222 (5)	88 (4)	60 (6)	28 (6)	46 (6)
**Chronic kidney disease, n (%)**	210 (5)	94 (5)	58 (6)	24 (5)	34 (4)
***Laboratory findings at admission***					
**Procalcitonin (ng/ml), median (p25–75)**	0.28 (0.11‒0.82)	0.3 (0.1‒1)	0.3 (0.1‒0.9)	0.3 (0.1‒0.8)	0.2 (0.1‒0.4)
**C-reactive protein (mg/dL), median (p25–75)**	14.19 (7.9‒22.3)	15 (9‒23)	15 (9‒23)	14 (7‒22)	6 (11‒17)
**White blood cells count (109/ml), median (p25–75)**	8.9 (6.3‒12.4)	10 (7‒13)	8.6 (6.3‒12)	8.2 (5.8‒11.5)	8.3 (5.0‒11.1)
**LDH (U/L), median (p25–75)**	489 (368‒670)	561 (434‒727)	512 (405‒629)	484 (384‒583)	409 (326‒507)
**D-dimer (ng/ml), median (p25–75)**	1000 (580.5‒2260)	1899 (813‒5659)	1128 (600‒2496)	991 (595‒2437)	837 (500‒1661)
**Creatinine (mg/dL), median (p25–75)**	0.76 (0.53‒1.03)	0.9 (0.7‒1.2)	0.8 (0.7‒1.1)	0.8 (0.7‒1.05)	0.8 (0.6‒0.9)
**Urea (mg/dL), median (p25–75)**	44 (32‒61)	47 (35‒67)	45 (32‒60)	44 (33‒57)	38 (29‒50)
**Lactate (mmol/L), median (p25–75)**	1.5 (1.1‒2.1)	1.1 (1.5‒2.1)	1.5 (1.1‒2.1)	1.6 (1.1‒2.3)	1.4 (1‒2.1)
**PaO**_**2**_**/FiO**_**2**_** ratio, median (p25–75)**	131 (90‒190)	155 (98‒215)	100 (75‒148)	116 (90‒152)	142 (106‒182)
***Complications and outcomes***					
**Acute renal failure, n (%)**	1123 (27)	676 (33)	283 (30)	111 (25)	53 (7)
**Renal replacement therapy, n (%)**	383 (9)	235 (12)	105 (11)	39 (9)	4 (1)
**Acute heart failure, n (%)**	376 (9)	208 (10)	104 (11)	42 (9)	22 (3)
**ICU LOS (days), median (p25–75)**^**b**^	14 (7‒28)^b^	19 (12‒34)	19 (12‒34)	25 (8‒42)	3 (5‒8)
**Hospital LOS (days), median (p25–75)**^**b**^	29 (18‒46)^b^	37 (25‒54)	35 (23‒53)	41 (20‒46)	15 (12‒21)
**ICU mortality, n (%)**	1270 (30)	787 (39)	293 (32)	163 (37)	0 (0)
**Hospital mortality, n (%)**	1327 (32)	824 (41)	309 (33)	176 (40)	0 (0)

*SOFA* Sequential Organ Failure Assessment, *APACHE* Physiology and Chronic Health Evaluation, *LOS* Length of stay, *LDH* Lactate dehydrogenase, *ICU* Intensive Care Medicine, *PaO*_*2*_*/FiO*_*2*_ Arterial oxygen pressure/ inspired oxygen fraction

^a^Measured after 24 h of admission

^b^Calculated on survivors

The conditional random intercept model found no effects on ICU mortality attributable to the centre (variance 0.39, SD 0.63; ICC 0.10) or to hospital size (variance 0.001, SD 0.04; ICC 0.0004). ICU mortality was 30% (*n* = 1260/4198) and hospital mortality was 32% (*n* = 1327/4198), with a median ICU length of stay (LOS) of 14 (7‒28) days and hospital LOS of 29 (18‒46) days. The complete characteristics of the whole population and groups are shown in Table 1.

At ICU admission, 2,024 (48%) patients were on IMV or immediately intubated (very early group). Additionally, 928 (22%) patients were intubated during the first 24 h after ICU admission (early group), 441 (10%) were intubated > 24 h after ICU admission (late group) and 805 (19%) were never intubated. Thus, a total of 3,393 (81%) received IMV during the ICU stay (Fig. 1). Very early intubation was more commonly performed in the first wave of the pandemic (56 vs 34%, *p* < 0.001) and patients never intubated were more common in the second wave (23 vs 17%, *p* < 0.001) (Table S1). Non-invasive respiratory support devices were used more often in the second wave (Table S2). Nevertheless, no significant differences in ICU mortality were observed between the first and second waves (31 vs 28%, *p* = 0.1).

Patients in the very early group were older and presented with a higher APACHE II and SOFA severity scores than the other groups. The crude ICU mortality in the very early group (39%) was higher than that observed in the early group (31%, *p* < 0.001) but similar to that of the late group (37%, *p* = 0.48) (Table 1).

### Early versus late intubation comparison

Patients in the late group were younger (62 vs. 64, *p* = 0.01), with less severe disease (APACHE II: 13 vs. 14, *p* = 0.007, and SOFA: 3 vs. 4, *p* < 0.001), and had higher PaO2/FiO2 ratio on ICU admission (116 vs. 100, *p* < 0.001). However, the mortality in the late intubation group (37%) was higher than the observed ICU mortality in the early intubation group (32%, *p* = 0.05) and hospital mortality in the late group was higher than in the early group (40 vs 33% respectively, *p* = 0.03, Table 2). There was no statistical difference in ICU (19 vs 20, *p* = 0.2) and hospital LOS (37 vs 35, *p* = 0.7) or days of IMV (20 vs 19, *p* = 0.99).

**Table 2:** Univariate analysis. Early vs. late intubation

**Variables**	**Early intubation (*n* = 928)**	**Late intubation (*n* = 441)**	***P* values**
***General characteristics***			
**Male, n (%)**	670 (73)	317 (72)	0.9
**Age (years), median (p25–75)**	64 (56‒71)	62 (50‒73)	0.01
***Comorbidities***			
**Hypertension, n (%)**	466 (50)	175 (40)	<0.001
**Obesity (> 30 kg/m**^**2**^**), n (%)**	354 (38)	150 (34)	0.1
**Diabetes, n (%)**	262 (28)	85 (19)	<0.001
**Chronic lung disease, n (%)**	69 (7)	24 (5)	0.2
**Asthma, n (%)**	60 (6)	26 (6)	0.7
**Immunosuppression, n (%)**	60 (6)	28 (6)	0.9
**Chronic kidney disease, n (%)**	58 (6)	24 (5)	0.5
**Chronic heart failure, n (%)**	32 (3)	18 (4)	0.5
**Ischemic heart disease, n (%)**	64 (7)	27 (6)	0.6
***Severity of illness***			
**SOFA, median (p25–75)**	4 (3‒7)	3 (2‒6)	<0.001
**APACHE II, median (p25–75)**^**a**^	14 (10‒17)	13 (10‒16)	0.001
**PaO**_**2**_**/Fio**_**2**_** ratio at admission, median (p25–75)**	100 (75‒148)	116 (90‒152)	<0.001
**Shock at admission, n (%)**	281 (30)	36 (8)	<0.001
***Laboratory variables***			
**Procalcitonin (ng/ml), median (p25–75)**	0.3 (0.1‒0.9)	0.3 (0.1‒0.8)	0.06
**C-reactive protein (mg/dL), median (p25–75)**	15 (9‒23)	14 (7‒22)	0.6
**White blood cells count (109/ml), median (p25–75)**	8.6 (6.3‒12)	8.2 (5.8‒11.5)	0.04
**LDH (U/L), median (p25–75)**	512 (405‒629)	484 (384‒583)	0,002
**D-dimer (ng/ml), median (p25–75)**	1128 (600‒2496)	991 (595‒2437)	0.1
**Creatinine (mg/dL), median (p25–75)**	0.8 (0.7‒1.1)	0.8 (0.7‒1.05)	0.1
**Urea (mg/dL), median (p25–75)**	45 (32‒60)	44 (33‒57)	0.5
**Lactate (mmol/L), median (p25–75)**	1.5 (1.1‒2.1)	1.6 (1.1‒2.3)	0.2
***Outcomes***			
**Days for IVM, median (p25–75)**^**b**^	19 (12‒34)	20 (12‒34)	0.99
**ICU mortality, n (%)**	293 (32)	163 (37)	0.05
**ICU LOS, median (p25–75)**^**b**^	20 (12‒35)	19 (12‒34)	0.2
**Hospital mortality, n (%)**	309 (33)	176 (40)	0.03
**Hospital LOS, median (p25–75)**^**b**^	35 (23‒53)	37 (24‒54)	0.7

*SOFA* Sequential Organ Failure Assessment, *APACHE* Physiology and Chronic Health Evaluation, *LDH* Lactate dehydrogenase, *IMV* Invasive mechanical ventilation, *ICU* Intensive care unit, *PaO*_*2*_*/FiO*_*2*_ Arterial oxygen pressure/ inspired oxygen fraction

^a^Measured after 24 h of admission

^b^Calculated on survivors

**Fig. 2:**
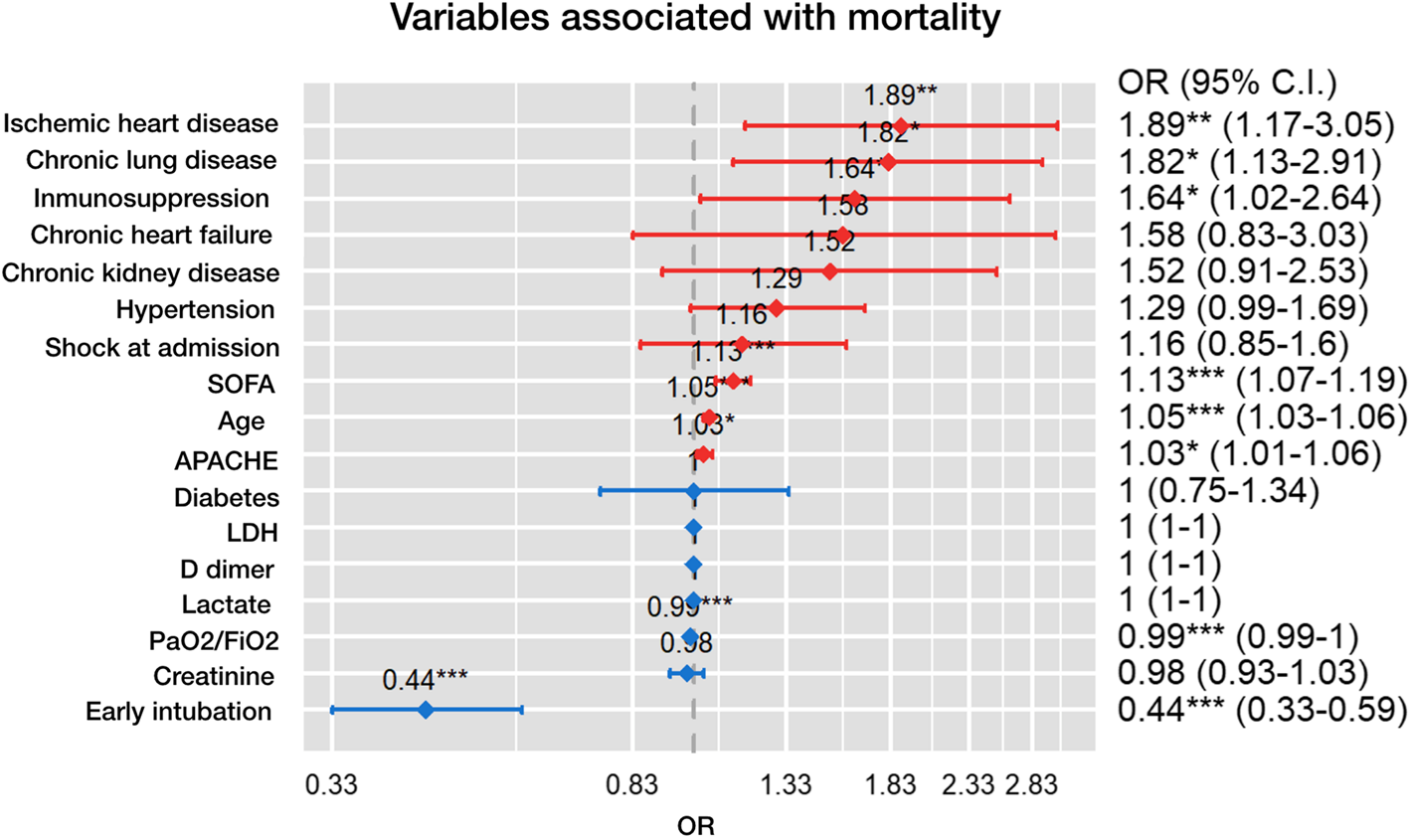
Variables associated with mortality. OR= Odds ratio, SOFA= Sequential Organ Failure Assessment, APACHE= Physiology and Chronic Health Evaluation, PaO_2_/FiO_2_ (arterial oxygen pressure/ inspired oxygen fraction), LDH: lactate dehydrogenase

Age, severity of illness (APACHE II and SOFA), hypertension, chronic kidney disease, COPD, diabetes, immunosuppression, coronary disease, and chronic heart failure were more frequent in non-survivors (Table S3) and were included in the multivariate model. Early intubation was found as an independent protective risk factor of mortality OR 0.44 (95%CI 0.33–0.59) (Fig. 2, Table S4).

To confirm these findings, propensity score (PS) matching was applied after excluding patients with less than 7 days of ICU LOS. 840 early patients and 371 late intubated patients were matched. The APACHE II score, SOFA score, age, presence of shock at ICU admission, diabetes, hypertension, LDH, PCT and PaO2/FiO2 ratio at ICU admission were the variables included in the PS model (Table S5). The summaries of balance for unmatched and matched data are shown in Table S6 and Fig. S1.

**Fig. 3:**
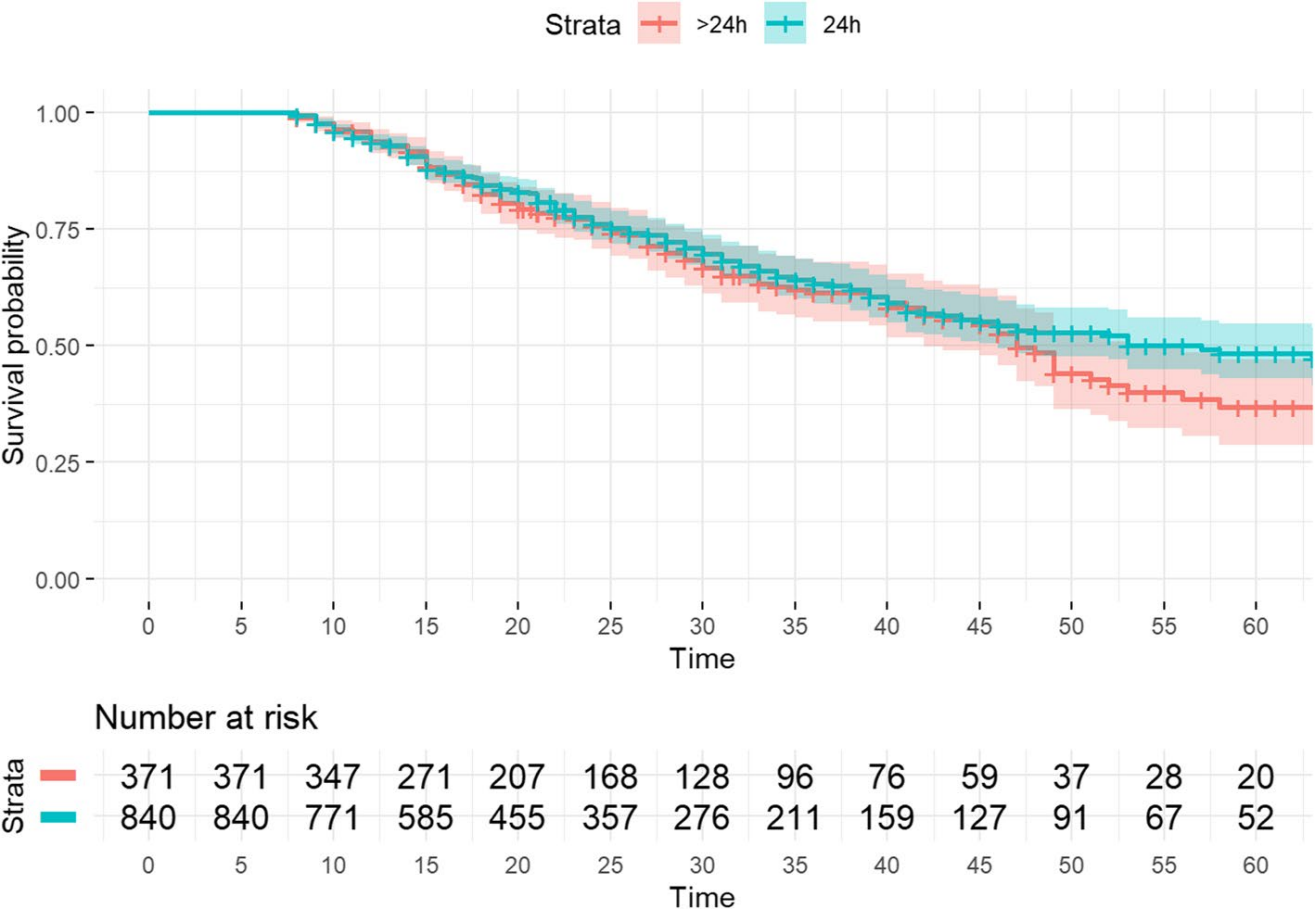
Cox regression ICU mortality

Finally, to determine the impact of early intubation on ICU mortality, a Cox regression analysis adjusted for potential confounding factors (Table S3) was performed in the matched cohort. The Cox model showed that the early intubation was significantly associated with a lower ICU mortality rate (HR 0.60; 95% CI 0.47-0.76) (Table 3, Fig. 3).

**Table 3:** Mortality in the ICU: Cox regression

**Variables**	**HR**	**IC**	**P values**
***General characteristics***			
**Age**	1.03	1.02‒1.05	<0.001
***Comorbidities***			
**Chronic lung disease**	1.3	0.9-1.8	0.2
**Chronic heart failure**	1.4	0.9-2.1	0.13
**Chronic kidney injury**	1.1	0.8‒1.8	0.5
**Diabetes**	1.1	0.9‒1.4	0.4
**Ischemic heart disease**	1.5	1.1‒2.1	0.01
**Hypertension**	1.1	0.9‒1.4	0.5
**Immunosuppression**	1.3	0.9‒1.8	0.1
***Severity of illness***			
**APACHE II**	1	0-9‒1.03	0.3
**SOFA**	1.1	1.02‒1.1	0.004
**Shock at admission**	0.88	0.7–1.2	0.4
**PaO2/FiO2 ratio**	0.99	0.99–0.99	<0.001
***Laboratory variables***			
**LDH (U/L)**	1	0.99–1	0.3
**D-dimer (ng/ml)**	1	1–1	0.2
**Lactate (mmol/L)**	1	0.99–1.002	0.1
**Creatinine (mg/dL)**	1	0.9.1.1	0.8
***Intubation timing***			
**Early intubation**	0.6	0.5‒0.8	<0.001

*APACHE* Physiology and Chronic Health Evaluation, *SOFA* Sequential Organ Failure Assessment, *PaO*_*2*_*/FiO*_*2*_ Arterial oxygen pressure/ inspired oxygen fraction, *LDH* Lactate dehydrogenase

## Discussion

The main finding of this research piece is that in critically ill patients with COVID-19 pneumonia who did not require intubation at admission to the ICU, intubation within the 24 h after ICU admission was found to be associated with a lower risk of mortality compared to those that were intubated out of the 24h window. Remarkably, we found higher mortality in the late intubation group compared to patients that were intubated within the first 24h even though those patients were younger, presented with less severe disease, and with a higher PaO2/FiO_2_ ratio at ICU admission.

The cut-off between early and late intubation remains controversial and somewhat arbitrary. Several studies using the cut-off 24-hours after ICU admission found no significant differences in ICU mortality [13, 18-21]. Studies using a 48-h cut-off have reported contradictory findings, with some observing higher in-hospital and out-of-hospital mortality rate in patients intubated after the cut-off [22] and others finding no significant differences [23, 24]. Both cut-off points were considered in the same sample in a prospective Spanish matched analysis study, finding increased mortality risk in both delayed intubation more than 24 and 48 h [25]. A retrospective observational study including only 40 patients with COVID-19 found lower mortality in patients intubated < 50 h after admission to ICU, but obviously the small sample could preclude conclusions about the timing of intubation [26]. Other prospective studies found an increase in mortality for each additional day between hospital admission and intubation [27, 28].

It might be more logical to establish the cut-off according to the onset of severe hypoxemic respiratory failure, although it will probably be coincidental to the ICU admission moment. Ranieri et al. [29] found no significant differences in mortality, ICU LOS, or days under IMV between patients intubated 24 h before or after the onset of ARDS diagnosed according to the Berlin criteria. However, another prospective study carried out in Spain, established the first 48 h after the start of any respiratory support as the cut-off point, and found a HR 2.2 for late intubation group [30].

When the decision to intubate is related with clear signs of ARF and late intubation is considered, early intubation is related with less mortality risk [31], as expected. In a study where patients were intubated if they developed hemodynamic instability, altered level of consciousness, or respiratory distress defined as the use of accessory respiratory muscles or inability to speak, Siempos et al. [32] found no significant differences in mortality, ICU LOS, or ventilator-free days. This study compared a group comprising non intubated patients together with those who were intubated after receiving non-invasive respiratory support for > 24 h to avoid intubation, versus the remaining intubated patients, probably because all the patients were intubated with clear signs of respiratory failure. A prospective observational study to assess the usefulness of a protocol in which patients were intubated for increased work of breathing after HFNC and awake prone manoeuvre found higher mortality in patients intubated > 48 h after ICU admission [33]. Finally, one of the latest meta-analyses published showed no statistical difference in terms of mortality between early and late intubation, however they analysed studies with different cut-off points and high heterogeneity, so the results must be interpreted with care [34]. The results obtained so far are contradictory, in part by the different cut-off points, wave patterns observed in the pandemic and intubation criteria in the studies analysed. More robust and protocolized studies, randomized, if possible, should be performed.

We established a 24 h after ICU admission cut-off point due to the characteristics of our database. Our findings were similar to those seen in patients with ARDS [35, 36] and some of the COVID-19 studies mentioned above, where delaying intubation increases mortality risk. This is supported by pathophysiology, as P-SILI is avoided when intubated due to decrease in respiratory drive and transpulmonary pressure [37]. It has been demonstrated that there are two phenotypes of COVID 19, type L with low elastance, low ventilation-perfusion ratio, low lung weight and low lung recruitment, and type H with high elastance, high lung weight and high recruitment. The second one could be the progression of the first one due to the illness evolution but also due to P-SILI [38].

Our study has several strengths. It is a multi-centre study conducted in 73 ICUs and including many critically ill patients. Moreover, data were collected over a 1-year period including different waves of the pandemic, thus avoiding possible biases related to variations in treatment arising from differences in the availability of resources and knowledge about the disease [16, 39, 40]. Finally, our robust statistical analysis adjusted for demographic variables, comorbidities, and severity scores and included propensity matched analysis.

Our study also has some limitations. The decision to intubate was not protocolized, and the reason for intubation was not recorded. Unfortunately, in order to obtain a large sample size with low missing data, we did not collect additional mechanical ventilation variables (e.g., driving pressure and mechanical power) [41]. Nevertheless, this epidemiological database compiled during the COVID-19 pandemic enables us to differentiate patients according to the timing of intubation in a real scenario. Our observational analysis carries the risk of selection bias, and other confounding factors cannot be excluded. For these reasons, despite the large number of critically ill patients included, our results may not be generalizable to other populations or scenarios.

## Conclusions

n COVID-19 patients, the decision to intubate within the first 24 h of ICU admission was associated with a protective benefit. It showed a lower risk of mortality compared to performing intubation after the first 24-h window of ICU admission. Our data suggest that physicians would consider a 24-h window to perform an intubation on COVID-19 patients admitted to ICU. Based on the observational nature of the data, this information should be validated further in future studies.

## Supplementary Information

**Additional file 1: Table S1.** OTI timing in different waves. **Table S2.** Oxygen-therapy at ICU admission differentiate by waves. **Table S3.** Univariate mortality analysis. **Table S4.** Mortality in the ICU: Binary logistic regression. **Table S5.** Early vs late group after excluding patients with < 7 days of ICU LOS. **Table S6.** Propensity score early and late groups. **Figure S1.** Propensity score early and late groups.

## Data Availability

The anonymized database and the data dictionary that defines each field in the set, will be available to reviewers if they consider necessary prior confidentiality agreement. Information contact: Alejandro Rodríguez, Critical Care Department – Hospital Universitario de Tarragona Joan XXIII, Tarragona, Spain, whose mail is ahr1161@yahoo.es.

## Abbreviations

ARDS: Acute respiratory distress syndrome
IMV: Invasive mechanical ventilation
VILI: Ventilator induced lung injury
P-SILI: Patient-self-induced lung injury
HFNC: High-flow nasal cannula
NIV: Non-invasive ventilation
APACHE: Acute Physiology and Chronic Health Evaluation
SOFA: Sequential Organ Failure Assessment
OR: Odds ratio
CI: Confidence interval
SD: Standard deviation
ICC: Interclass correlation coefficient
LOS: Length of stay
LDH: Lactate dehydrogenase
PaO_2_/FiO_2_: Arterial oxygen pressure/ inspired oxygen fraction
