# Supplementary material for: Timing of intubation and ICU mortality in COVID-19 patients: a retrospective analysis of 4198 critically ill patients during the first and second waves
Source: BMC Anesthesiol. 2023 Apr 27;23:140. doi: 10.1186/s12871-023-02081-5 (PMC10133910; doi:10.1186/s12871-023-02081-5)

**SUPPLEMENTARY MATERIAL**

Table S1: OTI timing in different waves.

| **Timing OTI** | **First wave**  **(N=2678)** | **Second wave (N=1520)** | **P values** |
| --- | --- | --- | --- |
| **Very early** | 1504 (56) | 520 (34) | <0.001 |
| **Early** | 510 (19) | 418 (27) | <0.001 |
| **Late** | 214 (8) | 227 (15) | <0.001 |
| **No OTI** | 449 (17) | 356 (23) | <0.001 |

OTI: Orotracheal intubation.

Table S2: Oxygen-therapy at ICU admission differentiate by waves.

| **Oxygen-therapy at admission** | **First wave**  **(N=2678)** | **Second wave (N=1520)** | **P values** |
| --- | --- | --- | --- |
| **IMV** | 1504 (56) | 520 (34) | <0.001 |
| **Conventional oxygen-therapy** | 574 (21) | 178 (12) | <0.001 |
| **HFNC** | 496 (18) | 691 (45) | <0.001 |
| **NIMV** | 104 (4) | 131 (9) | <0.001 |

IMV: Invasive mechanical ventilation, HFNC: high flow nasal cannula, NIMV: Non-invasive mechanical ventilation.

Table S3: Univariate mortality analysis.

| **Variables** | **Died in ICU (n=456)** | **Survived ICU stay (n=913)** | **P values** |
| --- | --- | --- | --- |
| ***General characteristics and severity of illness*** | | | |
| **Male sex, n (%)** | 343 (75) | 644 (70) | 0.08 |
| **Age (years), median (p25-75)** | 69 (62‒75) | 62 (53‒69) | <0.001 |
| **SOFA, median (p25-75)** | 5 (4‒7) | 4 (3‒6) | <0.001 |
| **APACHE II, median (p25-75)** | 15 (12‒19) | 13 (9‒16) | <0.001 |
| **PaO2/FiO2 ratio, median (p25-75)** | 101 (82‒125) | 115 (97‒132) | <0.001 |
| **Shock at admission, n (%)** | 125 (27.4%) | 192 (21%) | 0.01 |
| ***Comorbidities*** | | | |
| **Hypertension, n (%)** | 266 (58) | 375 (41) | <0.001 |
| **Obesity, n (%)** | 165 (36) | 339 (37) | 0.8 |
| **Diabetes, n (%)** | 142 (31) | 205 (22) | 0.001 |
| **Chronic lung disease, n (%)** | 54 (12) | 39 (4) | <0.001 |
| **Asthma, n (%)** | 27 (6) | 59 (6) | 0.8 |
| **Immunosuppression, n (%)** | 42 (9) | 46 (5) | 0.004 |
| **Chronic kidney disease, n (%)** | 45 (10) | 37 (4) | <0.001 |
| **Chronic heart failure, n (%)** | 29 (6) | 21 (2) | <0.001 |
| **Ischemic heart disease, n (%)** | 54 (12) | 37 (4) | <0.001 |
|  | | | |
| **Procalcitonin (ng/ml), median (p25-75)** | 0.32 (1.12-1.09) | 0.28 (0.12-0.78) | 0.14 |
| **C-reactive protein (mg/dL), median (p25-75)** | 14.9 (8.2-23.8) | 14.4 (8.3-22) | 0.35 |
| **White blood cells count (109/ml), median (p25-75)** | 8.7 (6.3-12.7) | 8.4 (6-11.7) | 0.12 |
| **LDH (U/L), median (p25-75)** | 528 (426-678) | 489 (382-589) | <0.001 |
| **D-dimer (ng/ml), median (p25-75)** | 1316 (700-3079) | 990 (555-2207) | <0.001 |
| **Creatinine (mg/dL), median (p25-75)** | 0.94 (0.74-1.25) | 0.8 (0.67-0.98) | <0.001 |
| **Urea (mg/dL), median (p25-75)** | 52.1 (41-69) | 40.1 (30-54) | <0.001 |
| **Lactate (mmol/L), median (p25-75)** | 1.8 (1.2-2.5) | 1.5 (1.1-2) | <0.001 |
| ***Timing of intubation*** | | | |
| **Timing of intubation** | | | 0.05 |
| **Early, n (%)** | 163 (36) | 278 (30) | <0.001 |
| **Late n (%)** | 293 (64) | 635 (70) | 0.87 |

ICU= Intensive Care Medicine, SOFA= Sequential Organ Failure Assessment, APACHE= Physiology and Chronic Health Evaluation, PaO2/FiO2 (arterial oxygen pressure/Inspired oxygen fraction), LDH= Lactate dehydrogenase.

Table S4: Mortality in the ICU: Binary logistic regression.

| ***General characteristics*** | | | |
| --- | --- | --- | --- |
| **Age** | 1.04 | 1.03‒1.06 | <0.001 |
| ***Comorbidities*** | | | |
| **Chronic lung disease** | 1.8 | 1.1-2.9 | 0.01 |
| **Chronic heart failure** | 1.6 | 1.8-3 | 0.2 |
| **Chronic kidney injury** | 1.5 | 0.9-2.5 | 0.1 |
| **Diabetes** | 1 | 0.7‒1.3 | 0.99 |
| **Ischemic heart disease** | 1.9 | 1.2-3.1 | 0.008 |
| **Hypertension** | 1.3 | 0.9‒1.7 | 0.06 |
| **Immunosuppression** | 1.6 | 1.02-2.6 | 0.04 |
| ***Severity of illness*** | | | |
| **APACHE II** | 1.03 | 1.006‒1.05 | 0.01 |
| **SOFA** | 1.1 | 1.06‒1.2 | <0.001 |
| **Shock at admission** | 1.2 | 0.8-1.6 | 0.3 |
| **PaO2/FiO2 ratio** | 0.99 | 0.98-0.99 | <0.001 |
| ***Laboratory variables*** | | | |
| **LDH (U/L)** | 1 | 0.99-1 | 0.1 |
| **D-dimer (ng/ml)** | 1 | 0.99-1 | 0.5 |
| **Lactate (mmol/L)** | 1 | 0.99-1 | 0.1 |
| **Creatinine (mg/dL)** | 0.98 | 0.9-1 | 0.5 |
| **Intubation timing** | | | |
| **Early intubation** | 0.44 | 0.32-0.59 | <0.001 |

APACHE= Physiology and Chronic Health Evaluation, SOFA= Sequential Organ Failure Assessment, PaO2/FiO2 (arterial oxygen pressure/Inspired oxygen fraction), LDH= Lactate dehydrogenase.

Table S5: Early vs late group after excluding patients with <7 days of ICU LOS.

| **Variables** | **Early intubation**  **(n=840)** | **Late intubation**  **(n=771)** | **P values** |
| --- | --- | --- | --- |
| ***General characteristics*** | | | |
| **Male, n (%)** | 601 (71) | 269 (72) | 0.8 |
| **Age (years), median (p25-75)** | 65 (56-72) | 62 (55- 69) | 0,008 |
| ***Comorbidities*** | | | |
| **Hypertension, n (%)** | 421 (50) | 154 (41) | 0,007 |
| **Obesity (>30 kg/m^2^), n (%)** | 323 (38) | 127 (34) | 0.2 |
| **Diabetes, n (%)** | 238 (28) | 70 (19) | 0,001 |
| **Chronic lung disease, n (%)** | 61 (7) | 16 (4) | 0.07 |
| **Asthma, n (%)** | 48 (6) | 21 (6) | 1 |
| **Immunosuppression, n (%)** | 54 (6) | 24 (6) | 1 |
| **Chronic kidney disease, n (%)** | 48 (6) | 15 (4) | 0.3 |
| **Chronic heart failure, n (%)** | 27 (3) | 17 (4) | 0.3 |
| **Ischemic heart disease, n (%)** | 57 (7) | 23 (6) | 0.8 |
| ***Severity of illness*** | | | |
| **SOFA, median (p25-75)** | 4.23 (3-7) | 4 (3-4.63) | <0.001 |
| **APACHE II, median (p25-75) a** | 14 (10-17) | 13 (10-16) | 0,001 |
| **PaO_2_/Fio_2_ ratio at admission, median (p25-75)** | 102 (88-120) | 127 (109-144) | <0.001 |
| **Shock at admission, n (%)** | 250 (30) | 27 (7) | <0.001 |
| ***Laboratory variables*** | | | |
| **Procalcitonin (ng/ml), median (p25-75)** | 0.3 (0.13-0.87) | 0.23 (0.1-0.7) | 0.02 |
| **C-reactive protein (mg/dL), median (p25-75)** | 15 (9-22.7) | 13.8 (7.4-21.6) | 0.06 |
| **White blood cells count (109/ml), median (p25-75)** | 8.60 (6.3-12.2) | 8.2 (5.7-11.4) | 0.04 |
| **LDH (U/L), median (p25-75)** | 510 (404-623) | 478 (382-573) | <0.001 |
| **D-dimer (ng/ml), median (p25-75)** | 1120 (597-2461) | 919 (580-2200) | 0.03 |
| **Creatinine (mg/dL), median (p25-75)** | 0.83 (0.69-1.08) | 0.84 (0.68-1.00) | 0.4 |
| **Urea (mg/dL), median (p25-75)** | 44.0 (31- 60) | 43 (33-56.5) | 0.45 |
| **Lactate (mmol/L), median (p25-75)** | 1.5 (1.1-2.05) | 1.5 (1.1-2.2) | 0.7 |
| ***Outcomes*** | | | |
| **Days for IVM, median (p25-75)*** | 21 (13-35) | 22 (14-35) | 0.35 |
| **ICU mortality, n (%)** | 245 (29) | 126 (34) | 0.11 |

APACHE= Physiology and Chronic Health Evaluation, SOFA= Sequential Organ Failure Assessment, PaO2/FiO2 (arterial oxygen pressure/Inspired oxygen fraction), LDH= Lactate dehydrogenase, IVM= Invasive mechanical ventilation, ICU= Intensive care unit.

Table S6: Propensity score early and late groups.

Summary of balance for all data:

|  | **Means treated** | **Means control** | **Mean difference** |
| --- | --- | --- | --- |
| **Distance** | 0.74 | 0.58 | 0.93 |
| **Age** | 62.96 | 61.42 | 0.13 |
| **No shock at admission** | 0.7 | 0.92 | -0.49 |
| **Shock at admission** | 0.29 | 0.07 | 0.49 |
| **No diabetes** | 0.71 | 0.81 | -0.21 |
| **Diabetes** | 0.28 | 0.18 | 0.21 |
| **No hypertension** | 0.49 | 0.58 | -0.17 |
| **Hypertension** | 0.5 | 0.41 | 0.17 |
| **APACHE II** | 14.21 | 12.94 | 0.22 |
| **SOFA** | 5.17 | 3.91 | 0.47 |
| **LDH at admission** | 566.55 | 501.64 | 0.17 |
| **Procalcitonin** | 1.42 | 3.22 | -0.21 |
| **PaO2/FiO2 at admission** | 106.58 | 133.48 | -0.7 |

Summary of balance for matched data:

|  | **Means treated** | **Means control** | **Mean difference** |
| --- | --- | --- | --- |
| **Distance** | 0.74 | 0.74 | 0.00 |
| **Age** | 62.96 | 61.81 | 0.09 |
| **No shock at admission** | 0.7 | 0.66 | 0.07 |
| **Shock at admission** | 0.29 | 0.33 | -0.07 |
| **No diabetes** | 0.71 | 0.72 | -0.01 |
| **Diabetes** | 0.28 | 0.27 | 0.01 |
| **No hypertension** | 0.49 | 0.53 | -0.06 |
| **Hypertension** | 0.50 | 0.46 | 0.06 |
| **APACHE II** | 14.21 | 13.84 | 0.06 |
| **SOFA** | 5.17 | 5.1 | 0.02 |
| **LDH at admission** | 566.55 | 533.64 | 0.08 |
| **Procalcitonin** | 1.42 | 3.07 | -0.19 |
| **PaO2/FiO2 at admission** | 106.58 | 107.79 | -0.03 |

HTA= arterial hypertension, APACHE= Physiology and Chronic Health Evaluation, SOFA= Sequential Organ Failure Assessment, PaO2/FiO2 (arterial oxygen pressure/Inspired oxygen fraction), LDH= Lactate dehydrogenase, IVM= Invasive mechanical ventilation, ICU= Intensive care unit, PCT= procalcitonin.

Figure S1: Propensity score early and late groups.


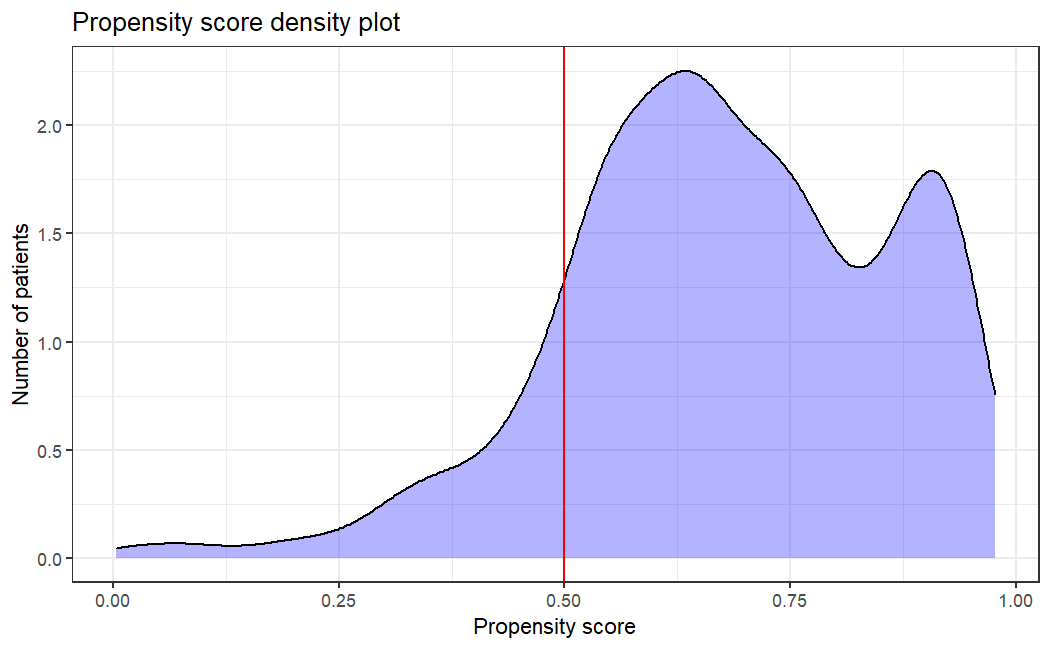

Supplement: Supplementary file 1 — Additional file 1: Table S1. OTI timing in different waves. Table S2. Oxygen-therapy at ICU admission differentiate by waves. Table S3. Univariate mortality analysis. Table S4. Mortality in the ICU: Binary logistic regression. Table S5. Early vs late group after excluding patients with < 7 days of ICU LOS. Table S6. Propensity score early and late groups. Figure S1. Propensity score early and late groups. [file 12871_2023_2081_MOESM1_ESM.docx]
